# ROCK Inhibitor Enhances Neurite Outgrowth In Vitro and Corneal Sensory Nerve Reinnervation In Vivo

**DOI:** 10.1167/iovs.65.12.31

**Published:** 2024-10-22

**Authors:** Beverly A. Karpinski, Sonali Pal-Ghosh, Himani Datta-Majumdar, Shelly Dimri, Soneha Datta, Mary Ann Stepp

**Affiliations:** 1 Department of Anatomy and Cell Biology, GW School of Medicine and Health Sciences, Washington DC, United States; 2 Department of Ophthalmology, GW School of Medicine and Health Sciences, Washington DC, United States

**Keywords:** cornea, epithelium, nerves, trigeminal ganglia, ROCK inhibitor, neurite outgrowth

## Abstract

**Purpose:**

The intraepithelial corneal nerves are essential to corneal health. Rho kinase or ROCK inhibitors (RIs) have been reported to play a role in neuron survival after injury. Here we assess integrin and extracellular matrix expression in primary mouse neurons and determine whether treating cells with RI impacts neurite outgrowth in vitro and reinnervation after trephine and debridement injury in mice in vivo.

**Methods:**

Cocultures of human corneal limbal epithelial cells and E11.5 mouse trigeminal neurons and neurons alone were grown on glass coverslips. High-resolution imaging was performed to localize integrins and laminin on neurons and to determine whether RI impacts neurite outgrowth in vitro and in vivo after both 1.5-mm trephine and 1.5-mm debridement injuries.

**Results:**

Several integrin α (α3, α6, αv) chains as well as β4 integrin are expressed on neuron axons and growth cones in cocultures. RI treatment of isolated neurons, cocultures, and in conditioned media increases neurite outgrowth. In vivo, RI positively impacts sensory nerve reinnervation after trephine and debridement injury.

**Conclusions:**

These studies are the first to demonstrate expression of β4 integrin on trigeminal sensory neurons and preferential adhesion of neurons to the laminin-enriched matrices found in footprints deposited by human corneal limbal epithelial cells. In addition, we also document for the first time the positive impact of RI on neurite outgrowth in vitro and reinnervation in vivo.

Rho kinase (ROCK) inhibitors (RIs) began to be used in ophthalmology clinics more than 15 years ago. Studies performed in the Shigeru Kinoshita group in Japan found that RIs enhanced corneal endothelial cell survival in vitro,^1^ allowing studies of human corneal endothelial cell physiology and function in vitro and improving our understanding of pathologies that impact the health of the corneal endothelial cells. For those studying the corneal epithelium, two studies on ROCK signaling published 16 years ago, one by the Nirmala SundarRaj group at the University of Pittsburgh,^2^ which showed that ROCK signaling regulated corneal epithelial cell proliferation and a second by the Fushin Yu group at Wayne State University in Detroit^3^ which showed that ROCK activation altered corneal epithelial cell differentiation and migration stimulating interest in ROCKs.

Several small GTPases signal to a serine/threonine kinase referred to as Rho-associated kinase or ROCK. ROCK signaling regulates cell migration via its ability to modify integrin mediated adhesion.^4^ Given the importance of cell adhesion to embryonic development and tissue integrity, as well as in wound healing and cancer, understanding how ROCK signaling works became imperative and led to the development and use of tools for its study. ROCK activity controls membrane protrusions at the leading edge of migrating cells by suppressing cytoskeletal remodeling, and RIs have been used as tools to understand the important roles played by integrins and the cytoskeleton in different cell types, including immune cells, keratinocytes, and neuronal cells.

The mouse cornea is innervated by a network of sensory nerves referred to as the subbasal nerve plexus or the intraepithelial corneal nerves (ICNs)^5^;^5^ ICNs consist of two components: intraepithelial corneal basal nerves that reside in the corneal epithelial basal cell layer between basolateral cell membranes, the basal cells, and the epithelial basement membrane; and the ICN terminals that branch from the intraepithelial corneal basal nerves toward the apical layers of the corneal epithelium. The ICNs have nerve cell bodies in the trigeminal ganglion. Because the majority of the ICNs enter the corneal epithelium from the corneal stroma at the corneal periphery near the limbus, when a 1.5-mm trephine is used to sever the ICNs, within 24 hours, 40% to 50% of the ICNs in the central cornea are lost.^6^^–^^8^ Although the trephine does not penetrate the stroma, anterior stromal nerve arbors are reduced 6 hours after trephine injury.^8^ ICNs grow back by extending their growth cones between intact desmosomes, adherens junctions, gap junctions, and hemidesmosomes. Despite these barriers, ICNs reinnervate quickly and within 4 days in healthy young mice axon density is restored.^7^ RIs have been reported to enhance corneal wound healing in mice,^9^ but little is known about the impact of RIs on the corneal sensory nerves and their elongation and reinnervation after trephine and debridement injury.

In this article, we develop methods that allow us to coculture corneal epithelial cells and trigeminal neurons to study the expression of integrins and laminin 332 (LN332) on trigeminal neurons and the ability of RIs to enhance neurite outgrowth on glass coverslips that have been coated with extracellular matrix derived from human corneal limbal epithelial (HCLE) cells. We then use RIs in an in vivo corneal trephine and debridement wound model to understand its impact on reinnervation of the male and female mouse cornea.

## Methods

### LN332-Coated Glass Coverslips

Fibronectin collagen type I (FN/CNI) solution was prepared as previously described.^10^ Glass coverslips were placed in six-well plates and coated with FN/CNI for 30 minutes at 37°C. FN/CNI was aspirated, and HCLE cells were plated on the coverslips and grown to 100% confluence in HCLE media. LN332-enriched matrix was prepared as described previously.^11^ In brief, freshly prepared 0.02 M ammonium hydroxide in 0.1% Triton-x100 was added to the wells with coverslips and left for 10 minutes at room temperature. Cellular debris was aspirated, wells and coverslips washed twice using the 0.1% Tx100 buffer followed by two washes with PBS.

### HCLE Cell Culture

Telomerase immortalized HCLE cells were generated^12^ and validated annually by STR analysis for the presence of mycoplasma. Stock vials of HCLE cells were thawed at 37°C and suspended in neutralization medium (500 mL of DMEM/F12 [Gibco #11039-021], 55 mL calf serum [Gibco #A3382001], and 5.5 mL 100X Pen-Strep [Gibco #15140-122]). Cells were then resuspended in HCLE media, which consists of supplemented GIBCO Keratinocyte SFM (Gibco #10724-011); the concentration of BPE used was 25 µg/mL (Gibco #13028-014) and of epidermal growth factor was 0.2 ng/mL (Gibco #10450-013) with 5 mL 100x Pen-Strep solution (Gibco # 15140-148). Cells were grown at 37°C with 5.0% CO_2_ and fed the next day and then every other day until used for experiments. Cells were not maintained in continuous culture; they were expanded as needed and frozen. For cell culture studies, RI (Y-27632; Tocris #1254) was used at 5 µM. The optimal concentration was determined from the literature^13^^,^^14^ and by titration of HCLE cells from 1 to 10 µM. Similar results were found using 5 µM and 10 µM; the lower concentration of 5 µM was used for these studies.

### Trigeminal Neuron Cultures

From 1 to 3 timed pregnant mice, 20 to 24 trigeminal ganglia were dissected from 10 to 12 E11.5 mouse embryos. As they were collected, ganglia were pooled in PBS in a microfuge tube and kept on ice. After all ganglia were obtained, the microfuge tube was spun for 1 minute at 1000 rpm, the PBS discarded, and 600 µL Digestion Media was added (10 mL Neurobasal Medium [Gibco #21103049], 20 µL 10% BSA [Hyclone # SH30574.01], and 10 µL Papain [Sigma # P3125]) and the ganglia were incubated at 37°C for 20 minutes. After incubation, the ganglia were microfuged for 1 minute at 1000 rpm, Digestion Media was discarded, and the pellet resuspended and triturated in 600 µL of L15 Complete Medium (L15 Medium [Gibco #11415064], 5% FCS [Gibco #A3382001], 0.02 M HEPES [Gibco #15630-080], and 1% Pen-Strep) until dissociated. The tube was then microfuged for 3 minutes at 1300 rpm, the L15 Medium discarded, and cells resuspended in Neurobasal Medium (96 mL Neurobasal Medium [Gibco # 21103049], 0.63 g glucose [Gibco # A16828.36], 2% B27 serum-free supplement [Gibco # 17504-044], 1% Glutamax supplement [Gibco #35050061], 1% Pen-Strep [Gibco, # 15140-148] and 50 µL human β-nerve growth factor [PeproTech #450-01]).

For cultures containing neurons only, HCLE LN332 coated coverslips were prepared in six-well plates as described above with one coverslip per well. Sterile cloning cylinders (12 mm top × 13 mm; SP BelArt #F37847-0300) were placed on the coverslips using a thin layer of sterile silicon grease (Sigma Aldrich # 85410). We placed 300 µL of neurobasal medium in the cylinder and, typically, 100 µL of neurons in neurobasal were plated into the cylinder. Neurons were given 1 to 2 hours to adhere before removing media and adding fresh neurobasal media, media supplemented with 5 µm/mL RI, or with neurobasal conditioned media (nbCM). Wells were filled with the same media as in the cylinder so that each well of the six-well plate contained one coverslip with its cloning cylinder. Neurons were incubated at 37°C with 5% CO_2_ for 24 hours. For cocultures, 100 µL neurons in suspension were added to subconfluent HCLE cells grown on coverslips in six-well plates. HCLE cells were grown in HCLE media until neurons were to be added; then media was changed to Neurobasal Media prepared as described elsewhere in this article.

### CM Preparation

**Figure 1.**
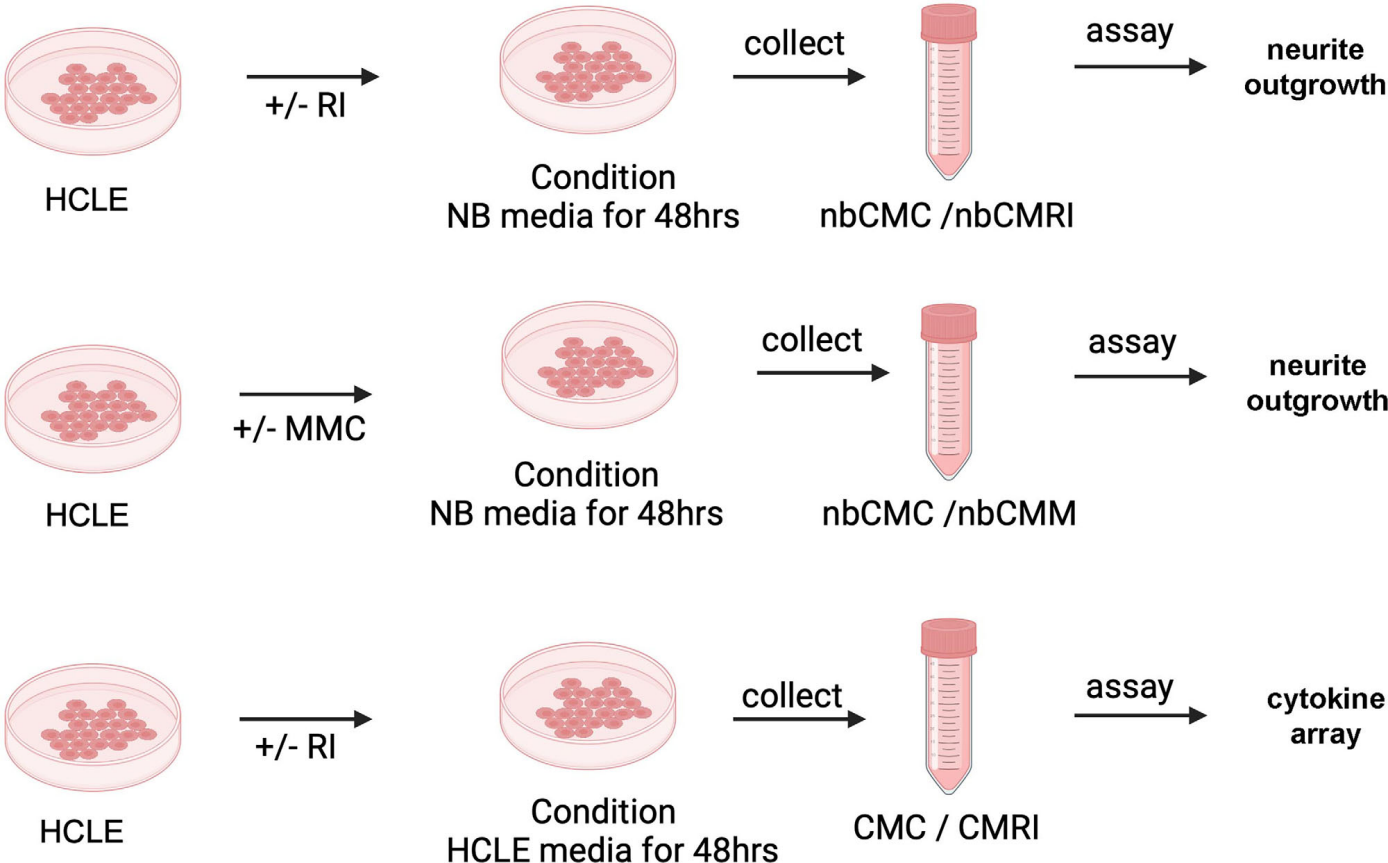
Preparation of CM. Schematic describes the procedure used to prepare CM for neurite outgrowth and cytokine array studies. Image prepared using Biorender.

CM was prepared from HCLE cells. A schematic for how CM was prepared is shown in Figure 1. HCLE cells were plated out in HCLE media in 100-mm^2^ dishes. Fresh media was added after 24 hours with or without 5 µM RI. At 24 hours, cells were washed to remove RI and shifted to neurobasal media for 48 hours. Debris was removed by centrifugation at 1000 rpm for 4 minutes and the number of cells that generated the CM was determined and used to normalize the volume of the CM and then frozen at −80°C. CM from control corneal epithelial cells is referred to as nbCMC and CM from RI-treated epithelial cells as nbCMRI.

### Cytokine Assays of CM

Conditioned media from control untreated cells (CMC) and condition media from RI treated cells (CMRI) were used for human cytokine antibody arrays (R & D Systems, #ARY005) as per the manufacturer's instructions. At least two different preparations of CM were used for on two arrays per variable and quantified using the Quick spots program from Ideal Eyes system.

### Immunofluorescence of HCLE and Trigeminal Ganglion Cells on Coverslips

Immunofluorescence was performed on HCLE cells after fixation in a mixture of 5% glutaraldehyde (Electron Microscopy Sciences #16120), 2% formaldehyde (Electron Microscopy Sciences #15712), in 0.1 M Na cacodylate buffer (Electron Microscopy Sciences #11654) for 30 minutes at room temperature. Fixative (2× concentration) was added 1:1 to the cell media followed by permeabilization using Triton-x 100 as described previously.^15^ Images were acquired at 100× magnification with an oil immersion lens using a Nikon Eclipse TS2R fluorescence microscope. The following antibodies were used: α3 integrin (1:200),^16^ α6 integrin (1:200; GoH3 Santa Cruz Biotechnology #sc19622), laminin-332 (1:500; LN332; J18),^11^ LAMC (1:200; Invitrogen #PA521514) αv integrin (1:200; BD Pharmingen #552299), β4 integrin (1:200; BD Pharmingen #553745), βIII tubulin (1:200; Tuj1; Biolegend #801201), and f-actin was visualized using phalloidin (Molecular Probes/Invitrogen #A12379 [green 488] and #A12381 [red 594]). Species-specific Alexa-fluor secondary antibodies (488, 594, and 647; Jackson Immunosciences) were used at 1:500 dilution in blocking buffer. Images were acquired using Nikon Eclipse TS2R and quantified using NIS Elements BR v5.00.

### Neurite Outgrowth Measurements

Cultures were fixed as described in the Methods section 24 hours after having been plated out and images of individual neurons were acquired at 20× magnification using a Nikon Eclipse TS2R fluorescence microscope. Axon lengths were traced and measured using Image J. A minimum of 50 axons were measured per variable assessed and experiments were repeated three times.

### In Vivo Trephine and Debridement Wound Healing

All studies performed were approved by The George Washington University Institutional Animal Care and Use Committee guidelines and in compliance with the ARVO Statement for the Use of Animals in Ophthalmic and Vision Research. For these studies, 7- to 8-week BALB/c mice were ordered from Charles River Laboratories. Mice were anesthetized with ketamine/xylazine. Proparacaine topical anesthetic was applied to their ocular surface as described previously.^7^ Wounding was bilateral. Mice were treated either with 25 µL of PBS vehicle or 25 µL of RI at 100 µM for 3 minutes, after which excess liquid was removed with a kimwipe and erythromycin ointment was applied. For trephine wounds, a 1.5-mm dulled trephine was applied gently to the ocular surface and rotated once (180°), leaving a visible depression on the ocular surface that defines the proximal (corneal periphery) and distal (toward the corneal center) sites. For debridement wounds, the epithelial cells within the 1.5-mm area defined by the trephine were removed using a dulled blade. Corneas from 10 male and 10 female (nulliparous) mice were assessed per variable studied. After injury, topical erythromycin ophthalmic ointment was applied to prevent infection and keep the ocular surface moist until anesthesia wears off and blinking resumes. Mice were treated with vehicle or RI at the time of wounding, 6 hours after wounding, and twice the following day. For trephine wounds, mice were sacrificed 4 days after initial wounding whereas for debridement wounds, mice were sacrificed 4 weeks after wounding. The concentration of RI used was determined empirically; we used a concentration 20-fold higher than that used in in vitro studies, which was 100 µM. After CO_2_ euthanasia and before enucleation, corneas were treated with Richardson stain, a vital dye that stained exposed basement membrane, and excess stain was removed with PBS. At the time of enucleation, eyes were separated into left and right as well as open or closed based on inspection of the eyes under a dissecting microscope for staining of the cornea with the dye. Eyes were fixed immediately as described below.

### Immunofluorescence Whole Mounts

All eyes were fixed immediately after enucleation in a paraformaldehyde-containing fixative (1× PBS, 1% formaldehyde, 2 mM MgCl_2_, 5 mM EGTA, 0.02% NP-40) for 1 hour and 15 minutes at 4°C, followed by two washes for 10 minutes each in 1× PBS containing 0.02% NP40 at room temperature. Tissues were then placed in 4:1 methanol:dimethyl sulfoxide for 2 hours at −20°C and then stored in 100% methanol at −20°C until used for whole-mount staining studies. The back of the eye was removed, and the retina, lens, and iris removed before staining. Tissues were transferred to a graded Methanol-TritonX-100 series (75%, 50%, and 25% methanol:TritonX-100 for 15, 15, and 10 minutes, respectively). All incubations were performed with gentle shaking and at room temperature, unless otherwise specified. The eyes were washed twice in PBS, for 30 minutes each, followed by incubation with blocking buffer for 2 hours. Blocking buffer was made as follows: To 100 mL 1× PBS, 1 g of BSA was added, the mixture was stirred for 10 minutes, 1 mL of horse serum was added, and the mixture was stirred for 1 additional minute.

The tissues were then incubated overnight with primary antibody against βIII tubulin (Tuj1; Biolegend #801201) diluted in blocking buffer at 4°C. The next day, the tissues were washed five times with PBS and 0.02% Tween 20 for 1 hour each, blocked for 2 hours, and then incubated with secondary antibody diluted in blocking buffer overnight at 4°C. The following day, eyes were washed three times with PBS and 0.02% Tween 20 for 1 hour each, followed by nuclear staining with 4,6-diamidino-2- phenylindole (DAPI) for 5 minutes, and washed with distilled water. To achieve the best flattening, the corneas were placed epithelial side-up with Fluoromount G mounting media (#17984-25; Electron Microscopy Sciences) and cover slipped.

### Measuring Axon Density

Confocal microscopy was performed at the GW Nanofabrication and Imaging Center at the George Washington University Medical Center. For axon density measurements we performed Sholl analysis. Images at 25× magnification were acquired using the Zeiss Cell Observer Z1 spinning disk confocal microscope (Carl Zeiss, Inc.), equipped with ASI MS-2000 (Applied Scientific Instrumentation) scanning stage with z-galvo motor, and Yokogawa CSU-X1 spinning disk. A multi-immersion 25×/0.8 objective lens, LCI Plan-Neofluor, was used for imaging, with oil immersion. An Evolve Delta (Photometrics) 512 × 512 EM-CCD camera was used as detector (80-ms exposure time). A diode laser emitting at 568 nm was used for excitation (54% power). Each eye was imaged at the vortex to obtain 3 × 7 tile montages. Zen Blue software (Carl Zeiss, Inc.) was used to acquire the images, fuse the adjacent tiles, and produce maximum intensity projections. The adjacent image tiles were captured with overlap to ensure proper tiling. All images were acquired using the same intensity settings. Sholl analysis was performed using ImageJ as described previously.^6^ Sholl analysis yields data on the number of times an axon crosses each radius in the target. The radii range from 5 to 75 µm and are 5 µm part; there are a total of 15 radii assessed per target; the total number of intersections is divided by the total number of radii. We use the term axon density to indicate the average number of intersections per target for each cornea assessed.

### Measuring Axon Thickness

Axon thickness was analyzed using Image J. Each image was magnified to its maximum and a line was drawn using the line tool to the length of 1 pixel. Set scale was selected in the Analyze tab and the number 1 entered as the distance in pixels and the known distance was set as 0.533 for 25× images. The pixel aspect ratio was set to 1 and the units set to micrometers. Then the Global box was selected. Next, still in the Analyze tab, select Set measurements and Area. With the image magnification at 400%, a line was drawn spanning the width of an axon. The axon thickness measurements were repeated for 35 different axons in the periphery of each image and 15 axons in the center of each image and measurements are transferred to excel. For each variable, 8 to 10 corneas were assessed.

### Stromal Nerve Arborization

Using the spinning disk microscope settings described above under the section describing how axon density is measured for performing Sholl analysis, 3 × 7 tile montages of 25× images were acquired below basal cell layer to avoid imaging the ICNs; imaging was performed until stromal nerves were no longer visible. Tiles were fused and maximum intensity projections were generated. Stromal nerve arborization was quantified using Neuron J, which is accessed within Image J. After opening each stromal nerve image, the image was converted to 8 bit and saved as a .jpg file. The Add tracing tool from the tool bar was used to draw along the length of all of the stromal nerves in each image; after tracing each nerve, the mouse is double clicked to end the tracing. The Measure tracing tool is then used to determine the length of the stromal nerves traced in micrometers. For each sex and variable, stromal nerves in five corneas were assessed.

### Statistical Analysis

Data were analyzed for statistical significance using GraphPad Prism. When recommended owing to differences in standard deviations, nonparametric tests were used. A single asterisk indicates *P* values between 0.01 and 0.05 and data that are significant, two asterisks indicate *P* values between 0.001 to 0.01 and that data are very significant, three and four asterisks indicate *P* values between 0.0001 to 0.001 and <0.0001 and indicate that data are extremely significant. When there are no asterisks provided or the letters *n*s are seen above a graph, it means the *P* values was ≥0.05 and the data are not statistically significant.

## Results

### Mouse trigeminal ganglion cells express several integrins including β4 integrin, L1CAM, and LN332 and preferentially adhere to the LN332 secreted by HCLE cells

We set out to develop a coculture model to study neuron: corneal epithelial cell interaction using mouse E11.5 trigeminal ganglion cells and the well-characterized human corneal epithelial cell line referred to as HCLE cells. Primary trigeminal ganglion cell cultures grow in a specialized media referred to as neurobasal media. Pilot studies showed that HCLE cells grew well in neurobasal media so we cocultured primary TG neurons and HCLE cells. TG neurons were plated onto HCLE cells growing on glass coverslips coated with a mixture of FN/CNI. Epithelial cells, including corneal and epidermal cells, adhere to surfaces primarily using α6β4 and α3β1 integrins and deposit a LN332-rich extracellular matrix.^17^ As cells migrate, they leave behind extracellular matrix and cell membrane fragments containing integrins; these sites are referred to as footprints.

**Figure 2.**
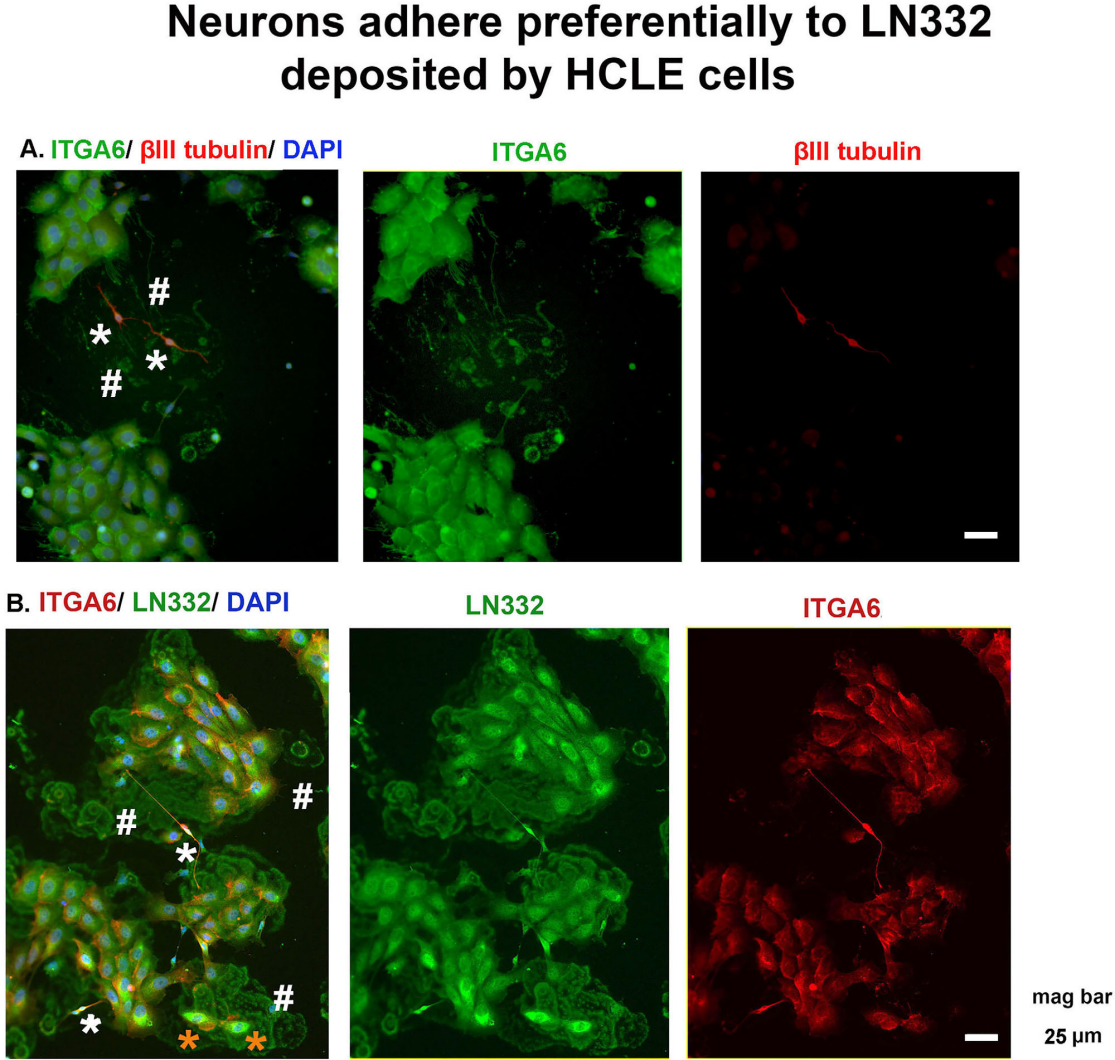
Neurons adhere preferentially to LN332 deposited by HCLE cells. (**A**) Representative images obtained from cocultures of neurons and HCLE cells fixed and stained to localize α6 integrin (ITGA6) (*green*), βIII tubulin (*red*), and DAPI (*blue*). Two neurons are shown (*) located at a region where ITGA6+ HCLE footprints are observed (#). The presence of DAPI+ nuclei in HCLE cells allows them to be differentiated from the footprints deposited as they migrate. (**B**) Representative images obtained from cocultures of neurons and HCLE cells fixed and stained to localize α6 integrin (*red*), LN332 (*green*), and DAPI (*blue*). HCLE cell LN332+ footprints are highlighted by the # and neurons and neural progenitor cells are shown by *white* and *orange asterisks*, respectively. In the images shown, α6 integrin and LN332 localize within axons, HCLE cell cytoplasm, and HCLE footprints. Scale bar, 25 µm

Shown in Figure 2A is a representative image of neuron: HCLE cocultures stained on the left for α6 integrin in green, βIII tubulin in red to reveal axons, and DAPI to show nuclei. Asterisks show two axons and the two # symbols highlight α6 integrin+ footprints. In Figure 2B, cocultures stained to localize α6 integrin and LN332 are presented. Footprints are positive for both α6 integrin and LN332. Note that axons seem to attach preferentially to surfaces at sites where HCLE footprints are located. Neurons are indicated by white asterisk and neuroprogenitor cells indicated by orange asterisks. Neurons express abundant levels of both α6 integrin and LN332 and adhere to sites on coverslips where LN332 matrix localizes.

**Figure 3.**
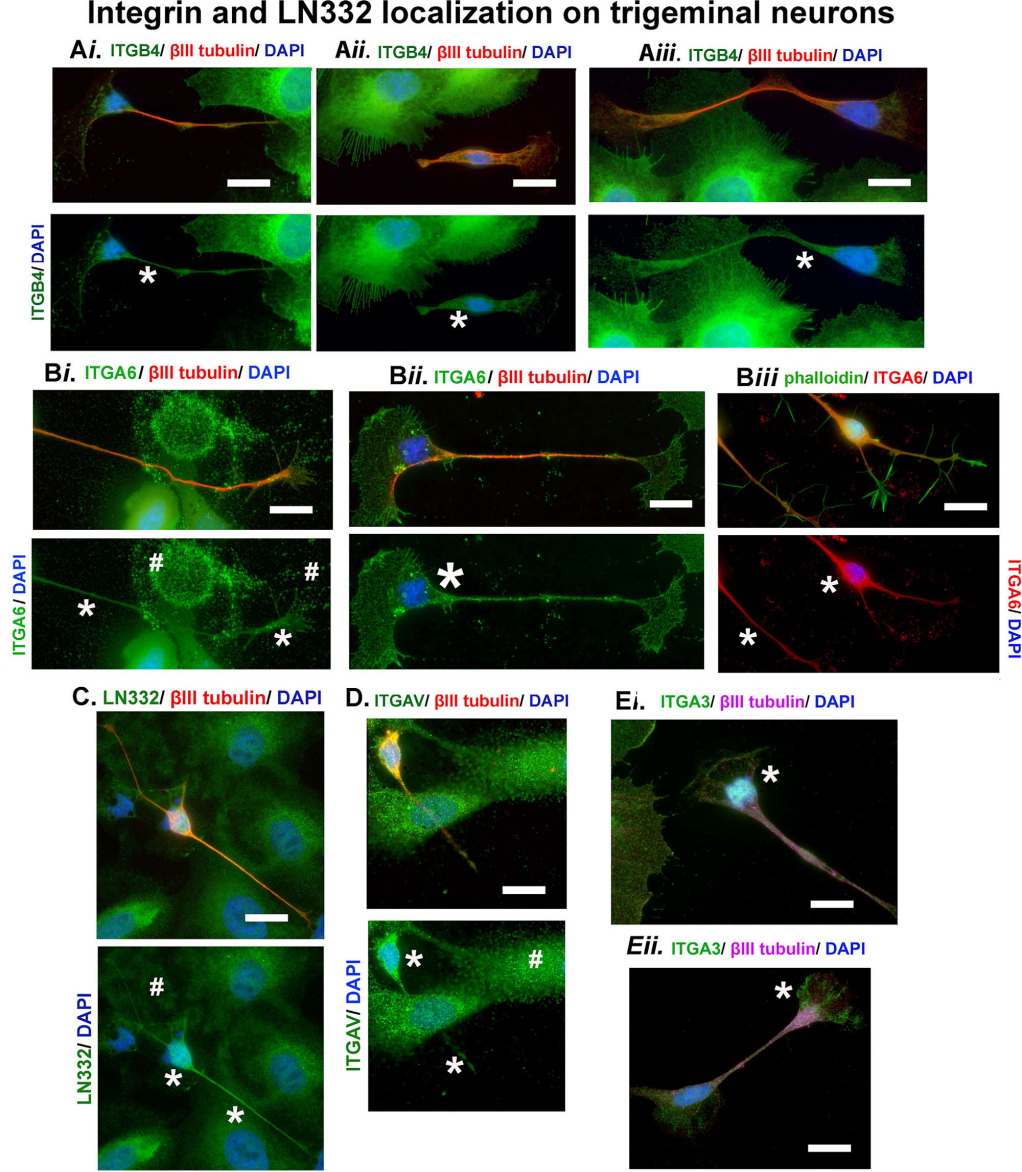
Neurons and HCLE cells express several of the same cell adhesion proteins. Representative images of neurons and HCLE cells cocultured and stained to visualize the following. (**Ai–Aiii**) β4 integrin (ITGB4) (*green*), βIII tubulin (*red*), and DAPI (*blue*). (**Bi and Bii**) α6 Integrin (ITGA6) (*green*), βIII tubulin (*red*), and DAPI (*blue* ). (**Biii**) f-Actin using phalloidin (*green*), α6 integrin (*red*), and DAPI (*blue*). (**C**) LN332 (*green*), βIII tubulin (*red*), and DAPI (*blue*). (**D**) αv integrin (ITGAV) (*green*), βIII tubulin (*red*), and DAPI (*blue*), (**Ei** and **Eii**) α3 integrin (ITGA3) (*green*), βIII tubulin (*magenta*), and DAPI (*blue*). Note that HCLE cells and neurons express α3, α6, αv, β4, and LN332. Neuron cell bodies and axons are indicated by * and footprints by #. Scale bar, 10 mm.

High-resolution images of axons visualized to show the localization of additional integrins are presented in Figure 3A through E. Images in 3A*i* through 3A*iii* show that trigeminal axons express β4 integrin. β4 Integrin is present within the cytoplasm near the axon nucleus as well as along the axon. As far as we can determine, ours is the first report of the presence of β4 integrin expressed in mouse trigeminal neuron cultures. Whereas α6 integrin can form heterodimers with either β1 or β4 integrin, β4 integrin form heterodimers exclusively with α6 integrin. Figures 3B*i* and B*ii* show high resolution α6 integrin staining along with βIII tubulin and DAPI; the α6 integrin+ axon in 3B*i* is navigating between an area containing an HCLE footprint and an HCLE cell expressing α6 integrin. Figure 3B*ii* shows a neuron cell body, axon, and growth cone; with the growth cone ending close to an HCLE cell. Figure 3B*iii* shows an axon stained with phalloidin to reveal f-actin–positive growth cones on axons in green with α6 integrin in red and DAPI in blue. The tips of the growth cone are enriched with f-actin, but contain little if any α6 integrin. In Figure 3C we confirm that axons express LN332. Figure 3D shows that axons express αv integrin, an integrin that forms heterodimers with multiple β subunits, including integrin β5 and β6 integrins in HCLE cells. Finally, Figure 3E*i* and 3E*ii* highlight axons in coculture expressing α3 integrin (ITGA3), which forms heterodimers with β1 integrins and mediates adhesion to various laminin isoforms. Like other integrins expressed in TG axons, α3 integrin localizes at the perinuclear region in the neuron cell body, along the axon, and near the growth cone.

The data presented in Figures 2 and 3 show an affinity of trigeminal neurons for laminin deposited by HCLE cells. Based on these data, for TG neuron culture experiments, we generated glass coverslips coated with laminin deposited by confluent cultures of HCLE cells to optimize our yield of TG axons. Intact HCLE cells are removed from the coverslips using ammonium hydroxide, a process that removes cells but leaves the matrix they deposited behind on the coverslip. The studies using neuron cell cultures and cocultures that follow were performed using laminin-rich HCLE matrix-coated glass coverslips.

### RI Increases Trigeminal Neurite Outgrowth In Vitro

**Figure 4.**
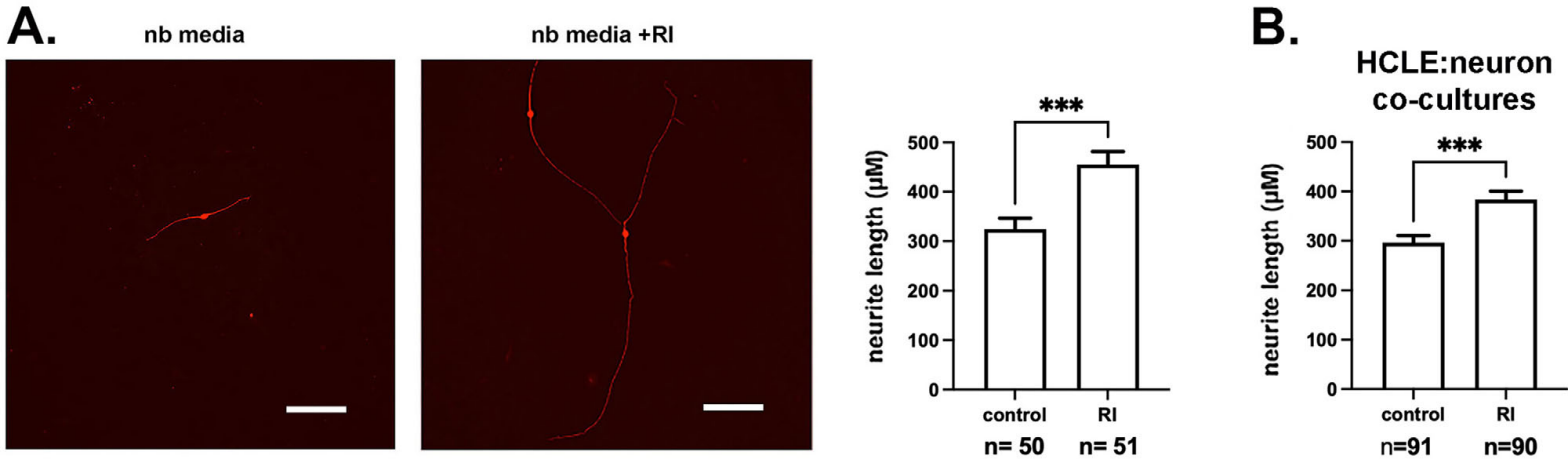
RI increases neurite outgrowth. (**A**) Neurite outgrowth is greater in TG neurons treated with RI compared with cells in control media**.** (**B**) Neurite outgrowth is greater in TG neurons cocultured with HCLE cells exposed previously to RI compared with coculture with untreated HCLE cells. Scale bar, 100 µm.

Trigeminal ganglia were dissected from E11.5 mouse embryos and grown overnight on glass coverslips that had been coated with laminin-rich HCLE-derived extracellular matrix (see Methods) in neurobasal media alone or in neurobasal media supplemented with RI. After fixation, neurons were visualized with an antibody against βIII tubulin and the lengths of neurites quantified. In Figure 4A, representative images of neurites are presented on the left and on the right, and quantification is shown. RI treatment increases neurite length significantly from a mean of 325 µm to 455 µm, which represents an increase of just under 30%.

### Coculturing Trigeminal Neurons With HCLE Cells That Had Been Previously Treated With RI Also Increases Neurite Growth

In vivo in the corneal epithelium, TG-derived axons are ensheathed by the corneal epithelial cells. To determine whether exposure of corneal epithelial cells to RI impacts their ability to support TG neurons, we next cocultured control and RI treated HCLE cells with trigeminal neurons. For these experiments, HCLE cells were allowed to grow overnight on glass coverslips in HCLE media. The next day, cells were fed with neurobasal media or neurobasal media supplemented with RI and cells allowed to grow overnight. Cells were then washed, and replaced with neurobasal media; TG neurons were added and cocultures grown in neurobasal media without RI. Data are presented in Figure 4B. HCLE cells exposed previously to RI enhance neurite growth to a mean of 380 µm compared with 300 mm for neurites growing with control HCLE cells. The increase in length for neurites cocultured with RI-treated HCLE cells is just over 20%.

### CM Derived From HCLE Cells Increases Neurite Growth

**Figure 5.**
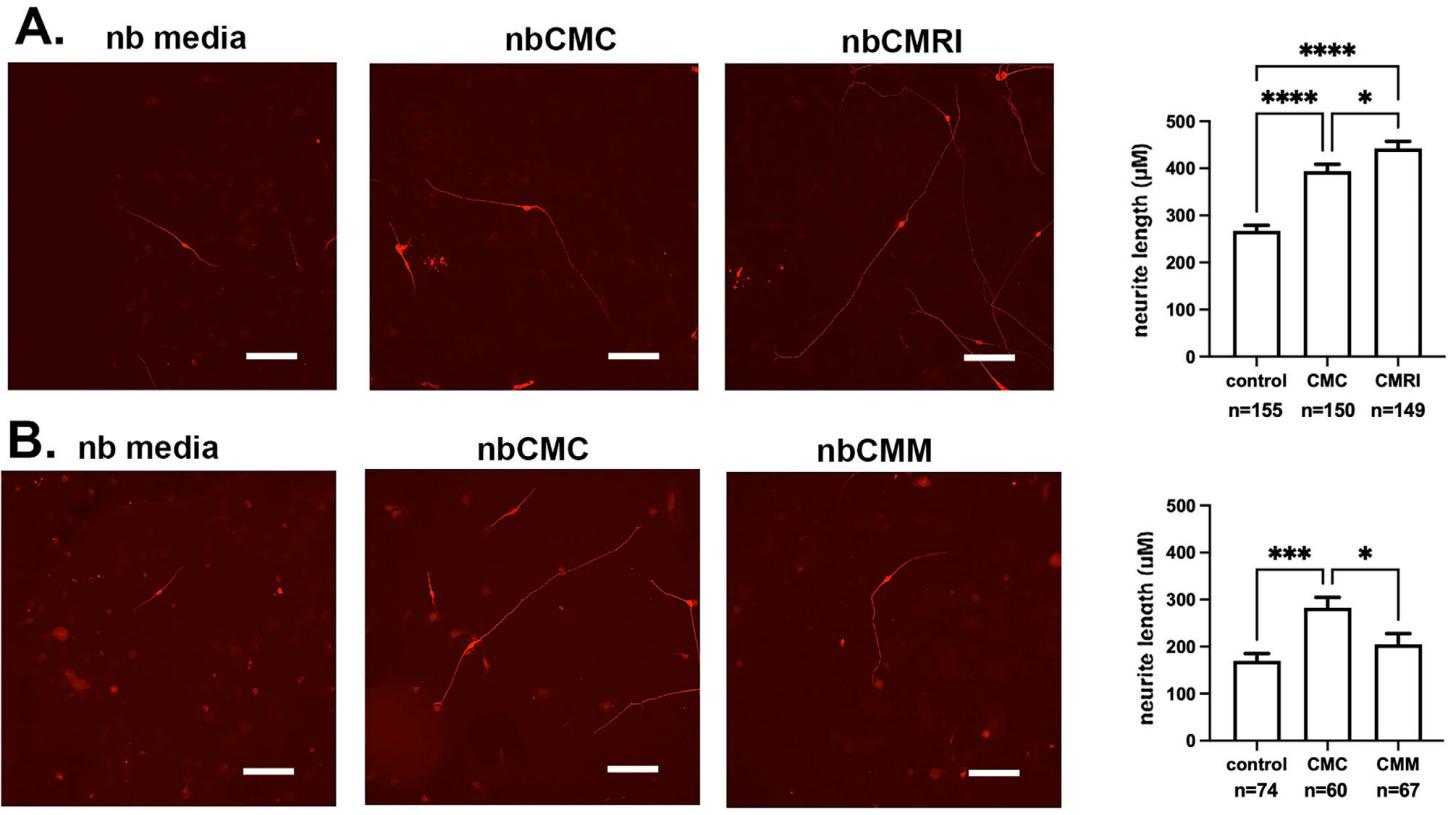
CM from RI treated cells increases TG neurite outgrowth. A description of the workflow used to generate the nbCM used for these experiments can be found in Figure 1. (**A**) Neurite outgrowth is greater in TG neurons cultured in CM from RI treated HCLE cells (nbCMRI) compared with TG neurons exposed to CM from control cells (nbCMC). (**B**) Neurite outgrowth is decreased in TG neurons cultured in CM from MMC treated HCLE cells (nbCMM) compared with TG neurons exposed to CM from control cells (nbCMC). Scale bars, 100 mm.

The results presented above indicate that RI treatment positively impacts neurite growth directly when added to neuron cultures and indirectly by exposure to RI-treated HCLE cells. Treated cells could either express proteins on their cell surfaces that enhance neurite outgrowth or could secrete factors into media that enhance neurite growth. To determine if the effects observed when neurites and RI-treated HCLE cells are cocultured requires direct contact between neurites and HCLE cells or is mediated by factors secreted by the HCLE cells, we next performed experiments using HCLE CM following the workflow described in the Methods section and Figure 1. Neurobasal CM (nbCM) from control nbCM (nbCMC), RI-treated nbCM (nbCMRI), and transiently mitomycin C (MMC)-treated nbCM (nbCMM) HCLE cells were generated and neurite outgrowth studies performed in primary TG neuron cultures. Neurons were grown in standard neurobasal media as well. Each study was replicated using a minimum of two separate preparations of CM. Data for nbCMC and nbCMRI neuron outgrowth studies are presented in Figure 5A and for control and transiently-MMC treated HCLE cells in Figure 5B. Representative images of neurites are shown on the left and quantification shown on the right. Previous studies by our group used CM derived from HCLE cells that had been transiently exposed to MMC showed that MMC treatment increased the release of cytokines that inhibit neurite outgrowth.^10^

Interestingly, nbCMC significantly increases neurite outgrowth compared with neurons grown in neurobasal media alone, but the extent of the increase can vary. For the data presented for the RI studies (Fig. 5A), nbCMC increases neurite growth from 270 µm to 390 µm or just over 30%; for the data presented for nbCMC for the MMC studies (Fig. 5B), nbCMC increases neurite growth from 170 to 280 µm or just over 40%. These data prove that corneal epithelial cells secrete factors that enhance neurite growth. nbCMRI further increases neurite growth, from 390 µm for nbCMC, to 440 µm for nbCMRI—just over 12%. By contrast, nbCMM decreases neurite growth from 280 µm for nbCMC to 205 µm for nbCMM, which is a decrease of just under 30%, resulting in neurite lengths similar to those seen in cultures with control neurobasal media. These results show that HCLE cells secrete factors into their media that enhance neurite outgrowth and that RI induces HCLE cells to secrete factors to further promote neurite outgrowth, whereas transient MMC treatment causes HCLE cells to secrete factors that interfere with neurite outgrowth.

### Cytokine Arrays of CM Secreted by Control and RI-treated HCLE Cells Show Changes in Only a Few Proteins

**Figure 6.**
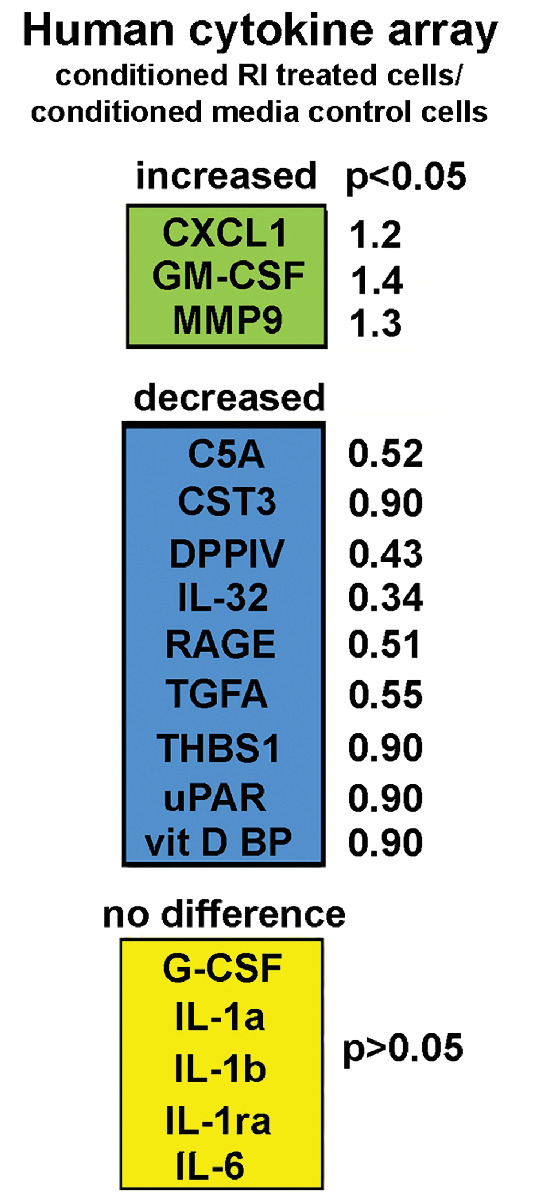
Cytokine array data showing changes in cytokine expression in CM from RI treated HCLE cells. The cytokines indicated in *green* were upregulated significantly and those shown in *blue* were downregulated significantly in CMRI compared with CMC. The cytokines shown in *yellow* are a subset of those that did not change in expression and had been shown previously to be upregulated in CM from transiently mitomycin C–treated HCLE cells

Previously, we used cytokine arrays to assess differences in the proteins released into CM by control HCLE cells and HCLE cells transiently exposed to MMC for 3 hours. We found nine cytokines that were increased significantly in expression in CMM including IL-1α, IL-1β, and IL-6^10^ and TGF-β1^18^ and none that were significantly decreased. Next, we used CMC and CMRI obtained from HCLE cells growing in HCLE media in cytokine arrays; results are shown in Figure 6. We found three proteins that were increased in CMRI compared with CMC: GM-CSF, CXCL1, and MMP9. All three proteins were also increased in CMM.^10^ We also found nine proteins whose levels were decreased in CMRI compared with CMC.

### Topical Treatment of the Mouse Cornea With RI Impacts Corneal Sensory Nerve Reinnervation After Trephine and Debridement Injury In Vivo

**Figure 7.**
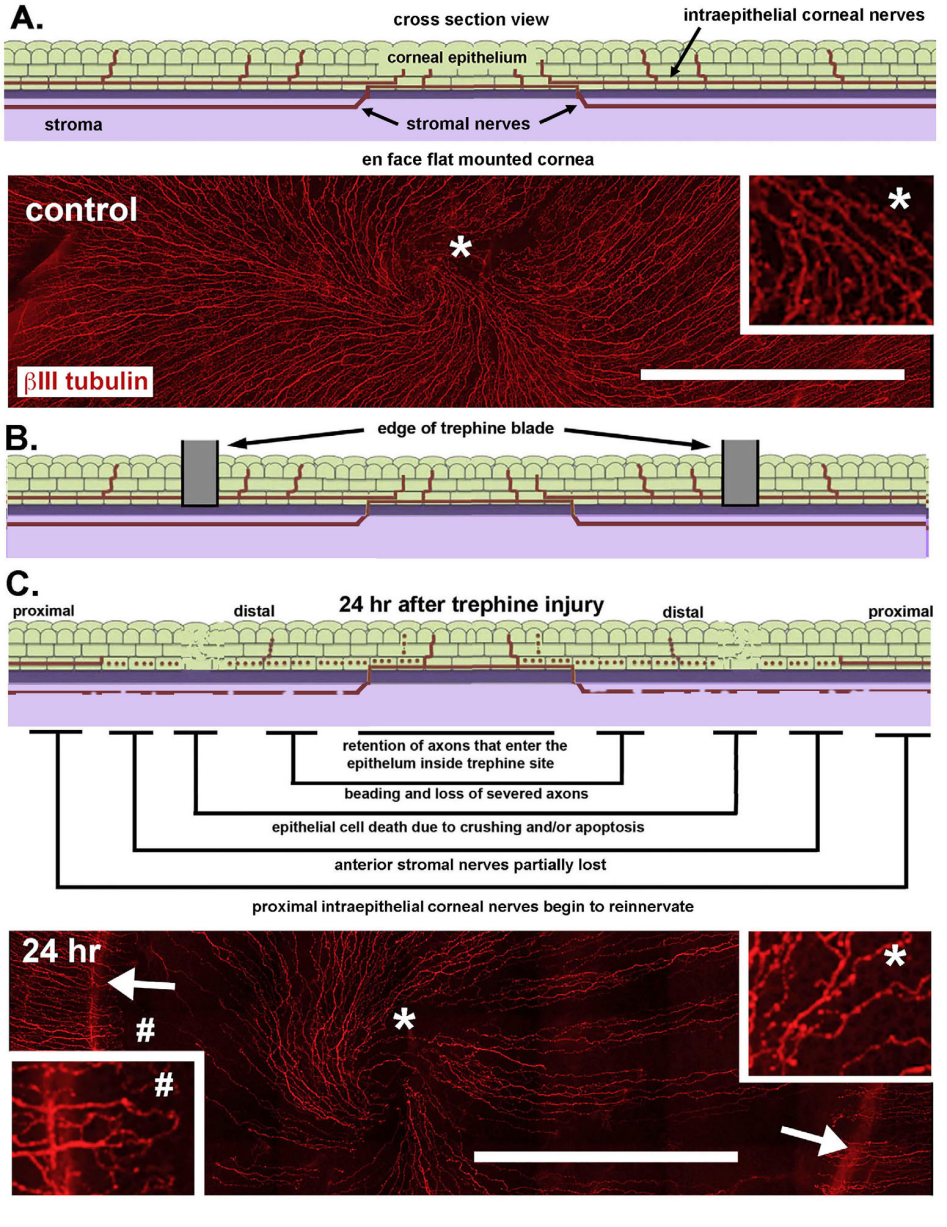
ICNs are partially denervated 24 h after trephine-only crush injury. (**A**) The schematic image at the top shows a cross-section of an unwounded cornea indicating the corneal epithelium, stroma, as well as the intraepithelial and stromal axons. Below is an enface view of an unwounded cornea stained with an antibody against βIII tubulin to show the ICNs. The site of the asterisk has been magnified digitally 4× in the inset. (**B**) The schematic image shows where the 1.5-mm diameter trephine blade comes in contact with the corneal surface and severs or crushes the axons and corneal epithelial cells. The trephine does not penetrate the epithelial basement membrane. The epithelium within the 1.5-mm trephine on the corneal surface remains alive and intact. (**C**) The schematic image above summarizes the changes that take place in the distal and proximal ICNs in the first 24 hours after trephine injury. Below is a representative enface confocal image of a whole flat mount mouse cornea showing ICNs 24 hours after trephine injury; the sites where the trephine blade came in contact with the corneal surface are indicated by *arrows*. The *inset* on the right shows the areas near the vortex indicated by the asterisk after being magnified digitally 4×. Compared with controls, fewer axons with more beading are present. Note that as early as 24 hours after injury, axons proximal to the injury site can be seen extending toward the corneal center. Bars in A and C, 500 µm. These images have been modified from a previous paper.^8^

The data presented herein support the hypothesis that corneal epithelial cells enhance TG neuron growth in vitro and that treating corneal epithelial cells and/or TG neurons with RI enhances neurite growth. To determine whether RI treatment impacts reinnervation in vivo, we used a well-characterized 1.5-mm trephine and debridement injury wound model. Figures 7A through C combines a schematic describing this model, as well as representative images of the intraepithelial cornea nerves in flat-mounted corneas stained with an antibody against βIII tubulin 24 hours after trephine injury. Near the corneal center, as shown in Figure 7A, the ICNs assume the form of a whirl that is typically referred to as the vortex. Although the number of axons in the vortex is decreased 24 hours after trephine injury, the vortex remains intact after trephine injury, as shown in Figure 7C.

We next treated male and female (nulliparous) Balb/c mice with PBS or RI (100 µm) at the time of 1.5-mm trephine injury followed by three more applications (one after 6 hours and two more 4 hours apart the next day). Mice are allowed to reinnervate their corneas for 4 days without further treatment and then euthanized. No epithelial cells are removed in this model; epithelial cell death injury is restricted to the site where the trephine blade penetrates the corneal epithelium. Previous studies have shown that, in this model, 40% to 50% of the axons within the circle defined by the 1.5-mm trephine disappear within 24 hours of injury and within 4 days, axon density recovers to levels similar to those seen in control mice.^7^ In addition, within 6 hours after injury there is a decrease in the numbers of immune cells in the corneal epithelial cells within the circle defined by the trephine.^8^

**Figure 8.**
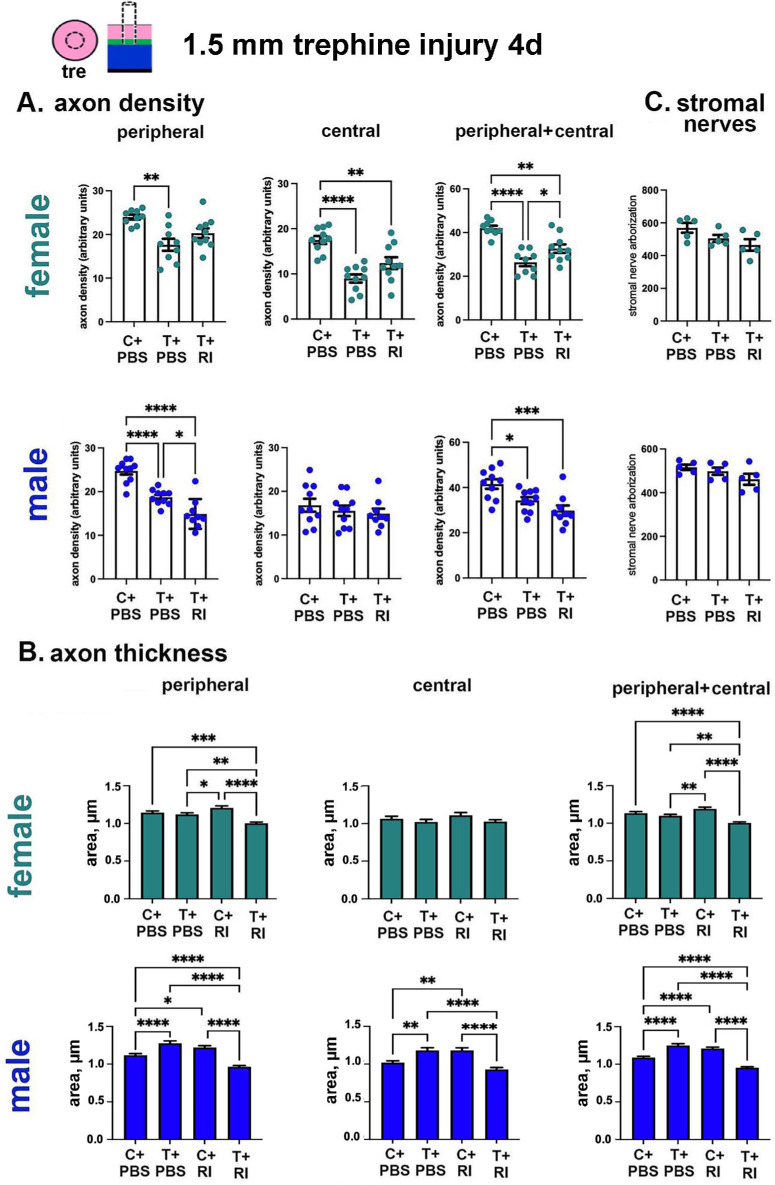
RI increases axon reinnervation in female but not male mice after trephine injury. Reinnervation of the corneal epithelial sensory nerves was studied at 4 days in PBS and RI-treated of unwounded (control) and PBS- and RI-treated trephine wounded corneas by assessing axon density and thickness. Data from female mouse corneas are highlighted in green and male data are shown in blue. (**A**) Axon densities are presented in the corneal periphery, central region, and in both periphery and central regions. Each filled circle represents a cornea. For each variable, 10 corneas were assessed. Outliers, determined by GraphPad Prism's outlier calculator, were excluded from the analyses with no more than one value excluded per variable. (**B**) The impact of RI treatment on axon thickness in male and female corneas before and 4 days after trephine injury were determined. Five corneas were assessed per variable with 35 axon diameters determined per cornea for the peripheral region and 15 axon diameters for the central cornea. Therefore, *n* was 175 for the peripheral data, 75 for the central cornea data, and 250 for the combination of central and peripheral. (**C**) Stromal nerve arborization in PBS control, PBS trephine, and RI-treated trephine injured male and female mice 4 days after trephine injury. Statistical significance was determined by ANOVA.

Corneas are fixed for whole mount confocal imaging and stained for βIII tubulin to allow us to assess axon density, axon thickness, and stromal nerve arborization; data are presented in Figure 8A through C. Although trephine injury primarily impacts the axons within the trephine site at the corneal center, reinnervation takes place from the corneal periphery as axons extend past the trephine site into the central cornea. Axon density and thickness at the periphery decreases, owing to axon branches retracting and/or increased axon bundling as they extend into the corneal center over time. For axon density and thickness, data are presented for the periphery, center, and for the combination of both values for female and male mice.

For female mice, shown in teal in Figure 8A, axon density at the periphery is reduced significantly after trephine injury compared with PBS-treated controls, and it is also decreased in the RI-treated trephine-injured corneal periphery, the decrease is not significant. In the central corneas of female mice, axon density is decreased significantly after trephine injury in the PBS- and RI-treated corneas; there is also a significant increase in the peripheral and central axon density in the RI-treated compared with PBS-treated trephine wounded corneas. As shown in Figure 8B, axon thickness decreases at the periphery, consistent with axons elongating and becoming thinner as reinnervation proceeds. At the corneal center in female mice, axon thickness for the trephine-injured corneas does not vary significantly compared with controls. Despite the increased number of axons growing into the RI-treated trephine-wounded corneal center compared with PBS-trephine–wounded corneas, those axons retain their thickness. In Figure 8C, we show that the decrease in stromal nerves caused by trephine injury was not significant in female PBS– or RI-trephine–injured corneas compared with controls.

For male mice, shown in blue in Figure 8A, at the periphery axon density is decreased significantly after both PBS- and RI-treated trephine injury compared with controls. However, peripheral axon density is decreased significantly more for RI-treated trephine-injured male corneas compared with its PBS-treated counterpart. In the central cornea, axon density is the same for all three variables indicating that the male mouse central cornea reinnervates its axons faster than female mice in response to trephine injury. The faster central corneal reinnervation in males for RI trephine injuries comes at the cost of axon density in the periphery with RI trephine–injured corneas having fewer axons than PBS trephine–wounded corneas and is not seen in females. When assessing the combined peripheral and central axon density data for the male corneas, unlike females, there is no significant difference between RI-treated compared with PBS-treated trephine-wounded corneas. After trephine injury in male corneas, shown in blue in Figure 8B, axon thickness after PBS-trephine injury increases at the periphery and at the corneal center, but decreases in the RI-treated trephine injured corneas at both sites. The increase in axon thickness in males after PBS-trephine injury is not seen after RI-trephine injury. Despite peripheral and central axon density for PBS- and RI-trephine–injured male corneas being similar, the axons growing into the male RI-treated trephine-wounded corneal center are thinner than those growing into the PBS-trephine–wounded corneas. In Figure 8C, we show that stromal nerve arborization was not changed significantly by injury for male PBS- or RI-treated trephine injured corneas compared with controls.

**Figure 9.**
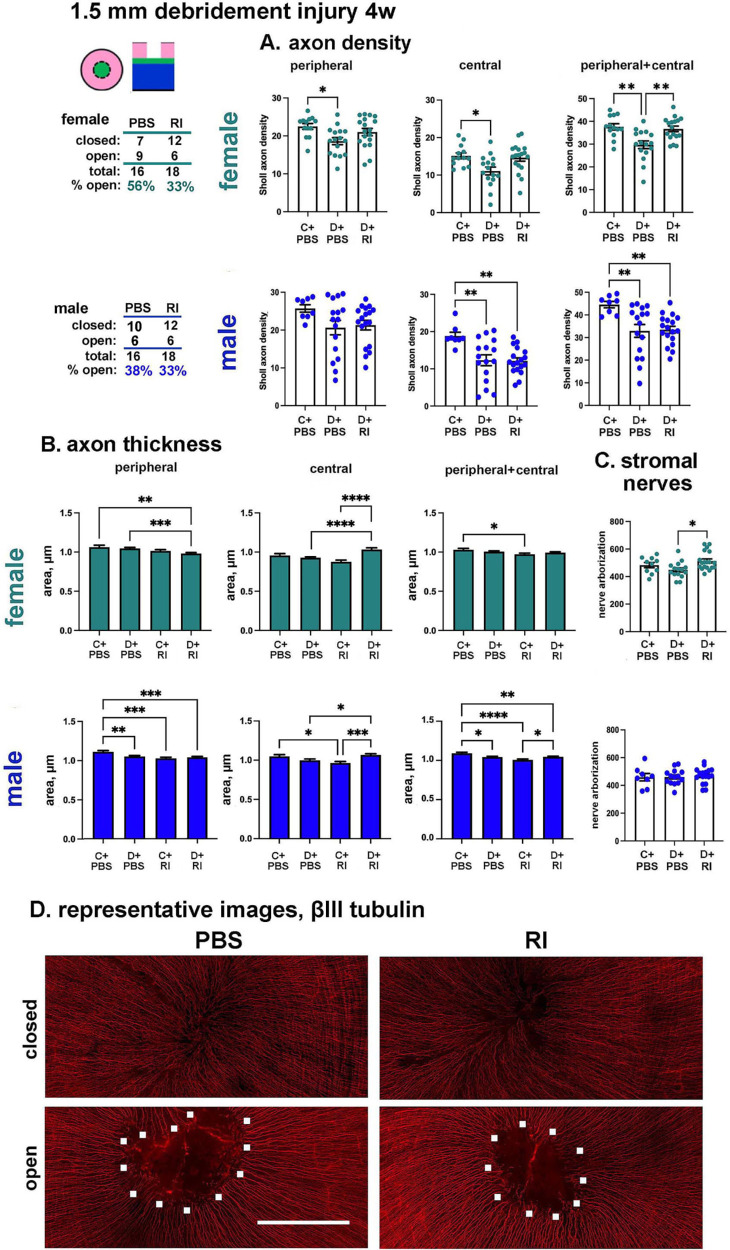
RI increases axon reinnervation in female but not male debridement wounded mice. Reinnervation of the corneal epithelial sensory nerves was assessed at 4 weeks in PBS- and RI-treated of unwounded (control) and PBS- and RI-treated female (*teal*) and male (*blue*) debridement wounded corneas by assessing ICN axon density and thickness, as well as stromal nerve arborization. After sacrifice, corneas were stained with Ricardson stain, which binds to the exposed basement membrane at erosion sites; after rinsing eyes with PBS, the presence or absence of erosions was assessed in a dissecting microscope. The numbers of open and closed corneas for vehicle- and RI-treated corneas is presented on the *upper left*. (**A**) Axon densities are presented in the corneal periphery, central region, and in both periphery and central regions. (**B**) The impact of RI treatment on axon thickness in male and female corneas before and 4 weeks after trephine injury was determined. Five corneas were assessed per variable with 35 axon diameters determined per cornea for the peripheral region and 15 axon diameters determined for the central cornea. (**C**) Stromal nerve arborization in PBS-control, PBS-trephine, and RI-treated debridement wounded corneas 4 weeks after wounding. Statistical significance was determined by ANOVA. Each filled circle represents a cornea. (**D**) Representative images from those used to obtain the data presented above are shown. *White squares* outline erosions sites in PBS- and RI-treated corneas. These images are representative of those presented in Supplementary Figure S1, which shows the corneas used for these analyses. Scale bar, 500 µm.

The 1.5-mm debridement wound was made using a dulled blade in a model that induce recurrent erosions in female and male mice 4 weeks after injury.^19^^,^^20^ At the time of wounding and the next day during reepithelialization, mice were treated 4× with 25 µL of 100 µM RI; control mice were treated with vehicle (PBS); at the time of euthanasia 4 weeks after debridement, corneas were stained to reveal the presence or absence of erosions via dissecting microscope and then fixed and stained for Sholl analysis, as well as for determination of axon thickness and stromal nerve arborization. Results for axon density, stromal nerve arborization, and axon thickness are shown in Figures 9A through 9C respectively for female (teal) and male (blue) corneas; representative images from female corneas 4 weeks after wounding are shown in Figure 9D. The vortex fails to reform even for corneas whose wounds are closed at the time of sacrifice. Tiled images for each cornea assessed for these debridement wound studies are shown in Supplementary Figure S1 for female corneas and Supplementary Figure S2 for male corneas.

For female mice, peripheral and central axon density, shown in teal in Figure 9A, are decreased significantly in PBS-wounded but not RI-wounded female corneas; the RI-treated wounded corneas have significantly greater axon density than the PBS-treated wounded corneas. In Figure 9B, we show that the axon thickness differences between variables 4 weeks after injury are minimal compared with what is seen 4 days after trephine wounding and presented in Figure 9B. There were differences in axon thickness between control and debridement-wounded corneas in the periphery 4 weeks after wounding, but in the central cornea, RI-treated wounded corneas have thicker axons than PBS-treated wounded corneas. Yet, when data are averaged over both peripheral and central regions, no wound-related differences are observed. However, axon thickness in the combined peripheral and central cornea is decreased significantly by RI treatment in control corneas compared with PBS control corneas. In Figure 9C, we show that RI-treated wounded female corneas have significantly greater stromal nerve arborization than the PBS-treated wounded corneas.

For male mice shown in blue Figure 9A, peripheral axon density is not altered significantly 4 weeks after wounding, but central axon density is significantly decreased in both PBS- and RI-wounded male corneas; however, unlike females, in male corneas RI-treated wounded corneas have similar axon densities as PBS-treated wounded corneas in the peripheral and central cornea. In Figure 9B we show that, although small, there are significant wound-related differences in the periphery of male corneas 4 weeks after wounding: axon thickness decreases in PBS-treated wounded corneas compared with PBS-treated controls, whereas it increases in RI-treated wounded corneas compared with RI-treated controls in the central and peripheral cornea. Of note is that the thickness of axons in control male corneas 4 weeks after RI treatment is decreased compared with PBS-treated controls. The decrease in axon thickness in RI-treated controls is seen in both female and male corneas 4 weeks after RI treatment. As shown in blue in Figure 9C, stromal nerve arborization is not impacted in male corneas by wounding.

To summarize these in vivo corneal wounding results, RI treatment enhances axon density significantly in two different corneal wound healing models in female but not male mice. Assessing axon thickness after trephine injury reveals sex– and RI-treatment–dependent differences in corneal nerves in response to trephine and debridement injury. Male and female corneas show that, after trephine injury, RI treatment decreased axon thickness significantly and PBS treatment increased axon thickness, but only in male corneas. After debridement injury, female axons show no differences in ,thickness whereas male axons are thinner in PBS-treated debridement-wounded corneas, but thicker in RI-treated debridement-wounded corneas. Whereas RI treatment of control corneas increased axon thickness as early as 4 days after treatment, the increase was only significant in male mice; in contrast, by 4 weeks after RI treatment, axon thickness was decreased both in males and females. By 4 days after trephine wounding, stromal nerves recover similarly in female, male, PBS-treated, and RI-treated corneas, whereas stromal nerve arborization is significantly greater 4 weeks after debridement injury for RI-treated female corneas.

## Discussion

### Trigeminal Neurons Express α6β4 Integrin and Prefer to Adhere to Laminin-rich Matrices Secreted by HCLE Cells

Here we present data describing how corneal epithelial cells function to provide glial-like support to sensory nerves in the corneal epithelium. Primary TG neurons prefer to adhere to the extracellular matrix deposited by HCLE cells and in our coculture model, TG neurons adhere preferentially to the surfaces of HCLE cells or on the footprints left by the HCLE cells. CM secreted by HCLE cells significantly enhances neurite outgrowth, indicating that corneal epithelial cells not only secrete matrix that support axons, but also secrete proteins or other molecules that allow neurons to grow longer.

We have shown previously that HCLE cells treated transiently with MMC causes the cells to secrete factors that interfere with corneal epithelial cell induced neurite outgrowth. CM from MMC treated HCLE cells contained elevated levels of proteins referred to as senescence associated secretory proteins, including several cytokines such as IL-1α, IL-1β, IL1-ra, and GM-CSF^10^;^10^ in cytokine arrays, none of the cytokines assessed were downregulated in CM from MMC treated cells. The contrast between the results obtained here with CM- from RI-treated cells and those reported previously on CM from MMC treated cells is striking. Only three proteins (CXCL1, GM-CSF, and MMP9) are upregulated in CM of RI-treated HCLE cells and nine are downregulated. The fact that more proteins are found to be decreased significantly in expression in CMRI compared with CMM suggests that RI treatment alters the balance between the secretion by HCLE cells of regeneration-associated proteins and proteins that inhibit axon growth by HCLE cells. However, the fact that one of the proteins upregulated is MMP9 suggests another potential mechanism for RI increased neurite outgrowth: a decrease in adhesion of the neurites to their substrate which could favor faster elongation. Another protein upregulated is GM-CSF, which is considered a proinflammatory cytokine that stimulates immune cell differentiation; it is also upregulated in expression in CM from MMC-treated HCLE cells. A recent study shows that GM-CSF enhances reinnervation in a mouse sciatic nerve crush wound model by enhancing infiltration of the nerve crush site with macrophages.^21^

Here we localize β4 integrin to the nerve cell body, axons, and growth cones of TG neurons. β4 integrin, which is known to function as an α6β4 heterodimers as an integral membrane component of hemidesmosomes, is also known to be expressed by Schwann cells; conditional deletion of β4 integrin in Schwann cells lead to decreased myelination of neurons in a sciatic nerve injury model. In the corneal epithelium, α6β4 is abundant on corneal epithelial basal cells and forms hemidesmosomes where it binds to laminin in the basement membrane; it is also present at basolateral locations in the basal cell layer, where the laminin secreted by the corneal epithelial cells ensheathes the intraepithelial corneal basal nerves in the basal cell layer. As a result, it has been impossible to determine with certainty whether the axons themselves express α6β4. In Supplementary Figure S3A and S3B, we provide evidence that the β4 integrin localizes to at least some of the axon nerve terminals in the suprabasal cell layers of the mouse corneal epithelium. Yet, we cannot exclude the possibility that apical epithelial cells retain α6β4 integrin exclusively at sites where they interact with nerve terminals. To confirm whether mouse TG neurons express β4 integrin, we have mined single nuclei RNA Seq data obtained from mouse TG ganglia. Supplementary Figure S3C shows that multiple different types of TG neurons express the mRNA for β4 integrin; these data have been confirmed in another single cell atlas of from mouse TG neurons.^22^ These new data validate data published in a separate DRG and TG ganglia atlas of mouse single cell RNA sequencing data.^23^

### RI Impacts Reinnervation In Vivo in Female Mice After Trephine and Debridement Wounds

The in vivo treatment of the mouse cornea with 100 µM topical RI 2× on the day of injury and 2× the next day after trephine or debridement injury improves axon density and thickness in female mice 4 days after trephine wounding, as well as 4 weeks after debridement wounding; similar differences are not seen in male mice. Female mice treated with RI reinnervated their corneas faster compared with vehicle treated corneas. Although axon density at the corneal center of male mice 4 days after trephine injury is the same as it is in control male and female mice, at the corneal center of female mice, axon density is significantly decreased compared with controls after both PBS-trephine and RI-trephine injury. Unlike the central region of RI-trephine–injured corneas of female mice, the central region of RI-trephine–injured corneas of male mice show decreased axon thickness compared with controls and PBS-trephine wounded–corneas. In female mice, axons that grow into the central cornea maintain their thickness. By contrast, in males, axons extend into the central cornea faster but do so by becoming thinner.

After debridement wounding, axon density is decreased compared with controls in PBS-treated female and male corneas, and in RI-treated wounded male corneas, but not in the RI-treated female wounded corneas where axon density is similar to controls. We first described a model for the study of recurrent erosions more than 20 years ago^19^ and have used it over the years to better understand the factors that control erosion formation.^6^^,^^8^^,^^18^^,^^24^^,^^25^ Although re-epithelialization is complete within 24 hours after wounding and ICNs have reinnervated the central cornea, the vortex never completely reforms and beginning 7 to 10 days after debridement, in healthy male and female mice, erosions begin to be observed. Over time, the erosions close and then reopen again at other sites on the cornea. The vortex never reforms. Control (unwounded) corneas treated with RI 4 weeks previously have thinner axons in both males and females; by contrast, unwounded corneas treated with RI 4 days before sacrifice had thicker axons in males but not females suggesting that the impact of RI on axon bundling does not require that the cornea be injured. In a recent 12-year study performed in patients by the Kinoshita group to study the ability of topical RI eyedrops to improve corneal endothelial cell function in patients with early stage Fuch's dystrophy, the same RI as in our study was used (Y-27632), but the concentration of the compound in the eye drops was 10 mM; RI improved clinical outcomes in patients and showed no signs of toxicity at that concentration.^26^ Based on this knowledge, it is likely that, had we used a higher concentration of RI for our experiments, we may have seen further enhancements in axon density. We use a lower concentration of 100 mM in our in vivo experiments to avoid toxicity; our concentration is 100-fold lower than what has been to treat patients successfully.

Using in vitro models, ROCK signaling has been implicated in regulating axon fasciculation.^27^ FIB-SEM^8^ and TEM^28^ studies have shown that the sensory axons visualized in whole flat mount corneas in the corneal epithelial basal cell layer are clusters of axons that include up to five to seven individual axons. When axons branch, axon thickness in the branches is decreased, making the axons more susceptible to breaking in response to mechanical forces owing to blinking and eye rubbing. We have shown previously that nerve terminals shed and regrow during homeostasis, and these events are subject to diurnal regulation. The exact roles that axonal thickness play in the stability of reinnervating ICN axons is not known.

In aging mice, we found that axon density was decreased by 80% at 24 months, but axon thickness remained the same as seen in young mice^29^;^29^ we suggested that bundling axons may protect aging axons from further loss. Yet, in two studies using genetically engineered mice, we found that decreased axon density and thickness can also occur together.^7^^,^^30^ Determining the exact relationship between axon density and thickness in the mouse cornea in response to injury will require additional research.

### Evidence for Sex Differences in Responses to RIs

Although there are numerous studies of RIs in the eye in animal models, most do not report data on male and female animals. We could find no research that evaluates the impact of sex on the ability of RIs to alter peripheral nerve regeneration. However, there are some data on the CNS and sex differences in RI responses. In a Parkinson's disease mouse model, the inhibition of ROCK delayed neuronal cell death via an estrogen-dependent mechanism involving decreased neuroinflammation.^31^ In a study of paclitaxel-induced hypersensitivity reactions in mice, male mice had a more robust mast cell activation response to paclitaxel than females^32^;^32^ the researchers went on to study the impact of RI treatment only in males and showed that RI decreased hypersensitivity via a mechanism involving nitric oxide. The activity of nitric oxide synthases has been reported to be regulated by female sex hormones.^33^^,^^34^

An elegant study by Pham et al.^35^ assesses re-epithelialization and reinnervation in several strains of young male and female mice after a 2-mm debridement injury performed using a rotating burr. They show significantly delayed wound closure times for female mouse corneas compared with males coupled with increased reinnervation times in females at 2 weeks after debridement injury. In addition, when they treat the corneas of male mice topically three times per day for 14 days with eye drops containing estrogen and injure the male mice while continuing the topical estrogen treatment for an additional 14 days, the male corneas treated with estrogen reinnervated their nerves significantly better than vehicle-treated corneas. These data indicate that reinnervation of the corneal sensory nerves after debridement is enhanced by female sex hormones. We found similar axon density in young male and female mice but, by 9 months of age, virgin (nulliparous) female mice had significantly decreased axon density compared with male mice^36^;^36^ however, this difference was eliminated in multiparous female mice, indicating that hormones secreted during pregnancy, which include estrogen and progesterone, delay age-related axon loss in the mouse cornea. Taken together, our studies comparing virgin and multiparous mice coupled with studies done by Pham et al. affirm that female sex hormones play roles in maintaining axon density both in response to injury and with age. The enhanced reinnervation of the RI-treated female cornea seen here may be due to differences in how male and female corneas respond to corneal injuries. More research needs to be done to work out the mechanisms that control these events.

## Conclusions

Here we use in vitro and in vivo models to explore ROCK signaling and its roles in regulating the interaction between axons and corneal epithelial cells. Our data indicate that ROCK signaling plays roles in regulating axon growth by functioning directly on neurons to enhance their elongation and indirectly by inducing corneal epithelial cells to secrete factors into their surroundings that enhance axon elongation. Whether the factors secreted by epithelial cells function as neuroprotective agents or direct axon elongation is not clear. Cytokine arrays suggest that subtle increases in MMP9, GM-CSF, and CXCL1 secretion by corneal epithelial cells play roles in this process. In the mouse cornea, inhibiting ROCK signaling can improve reinnervation after both trephine and debridement wounding. Additional studies are needed to understand the precise mechanisms controlling these events.

## Supplementary Material

Supplement 1
