# Supplementary material for: ROCK Inhibitor Enhances Neurite Outgrowth In Vitro and Corneal Sensory Nerve Reinnervation In Vivo
Source: Invest Ophthalmol Vis Sci. 2024 Oct 22;65(12):31. doi: 10.1167/iovs.65.12.31 (PMC11500046; doi:10.1167/iovs.65.12.31)
Supplement: Supplement 1 [file iovs-65-12-31_s001.pdf]

Supplemental Figures for:

ROCK inhibitor enhances neurite outgrowth in vitro and corneal sensory nerve reinnervation in vivo

Beverly A. Karpinski*, Sonali Pal-Ghosh*, Himani Datta-Majumdar*, Shelly Dimri*, Soneha Datta*, and Mary Ann Stepp*#

Funding Information: This work was supported by NIH/NEI EY08512 (MAS) and U01EY034692 (MAS). Imaging done in the GWU Nanofabrication and Imaging Center was funded by S0100D010710-01 and S0100D032420-01. The funders had no role in study design, data collection and analysis, decision to publish, or preparation of the manuscript.

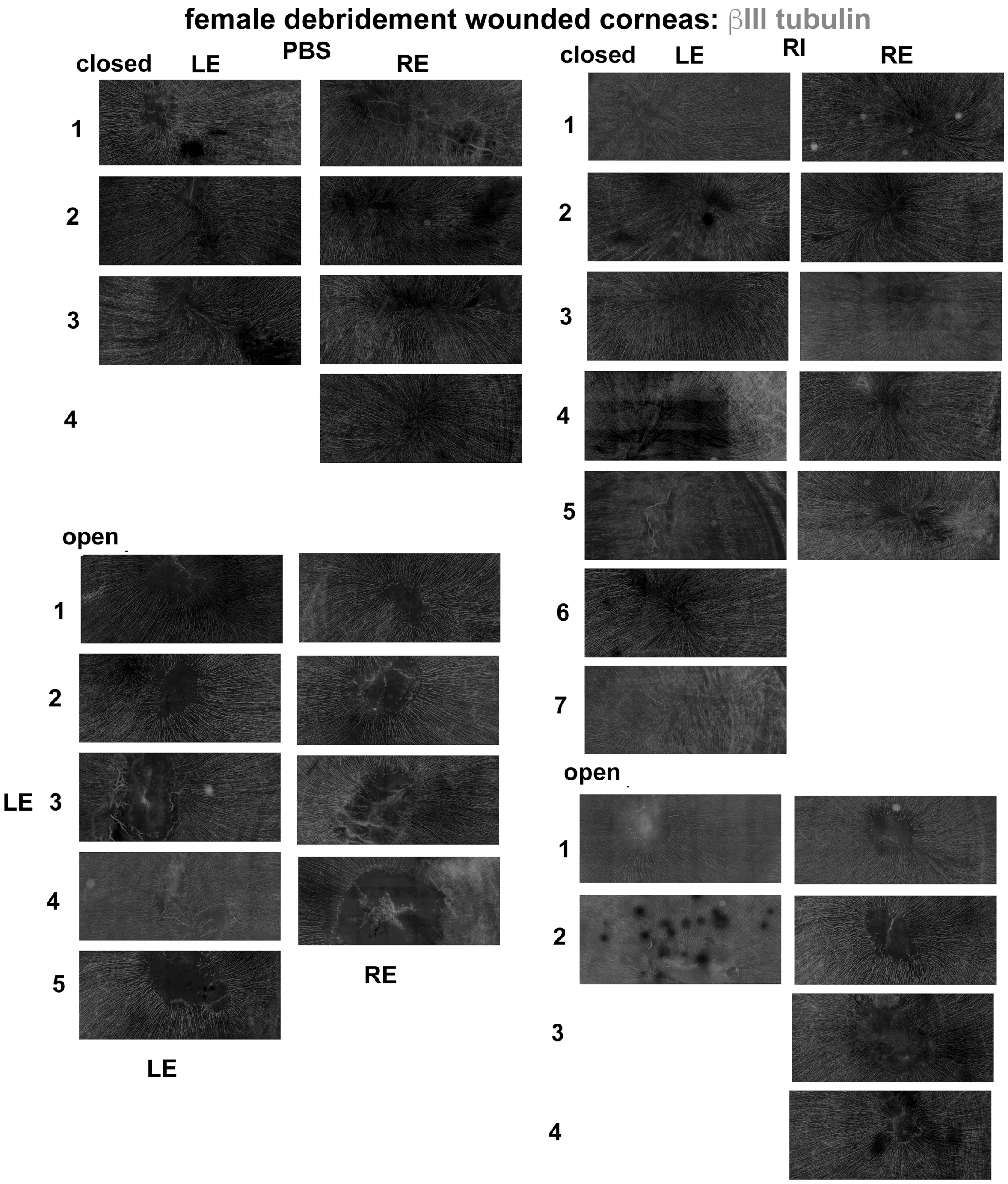

Supplemental Figure 1: Images of corneas used for Sholl analysis and axon thickness studies in female mice.

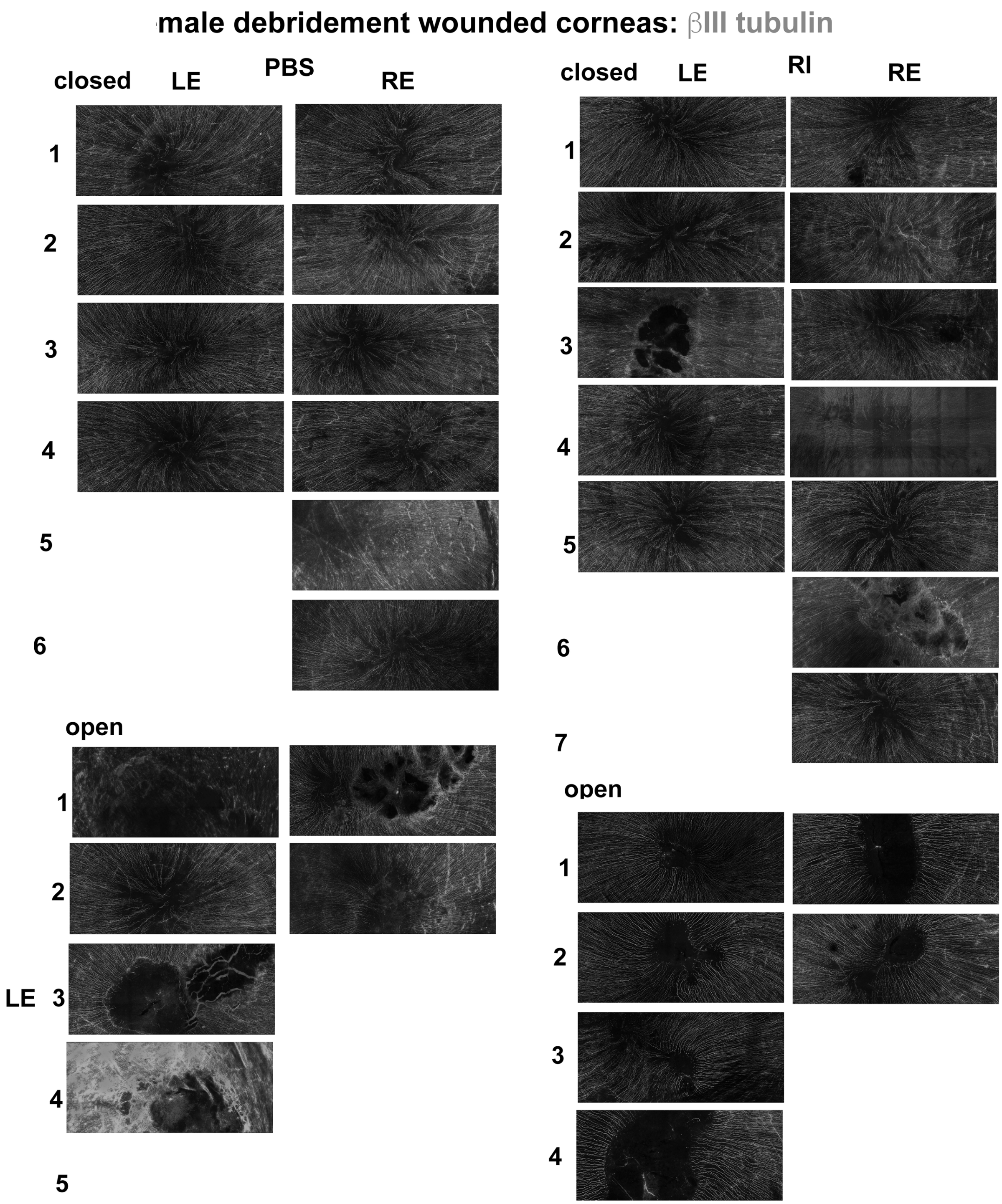

Supplemental Figure 2: Images of corneas used for Sholl analysis and axon thickness studies in male mice.

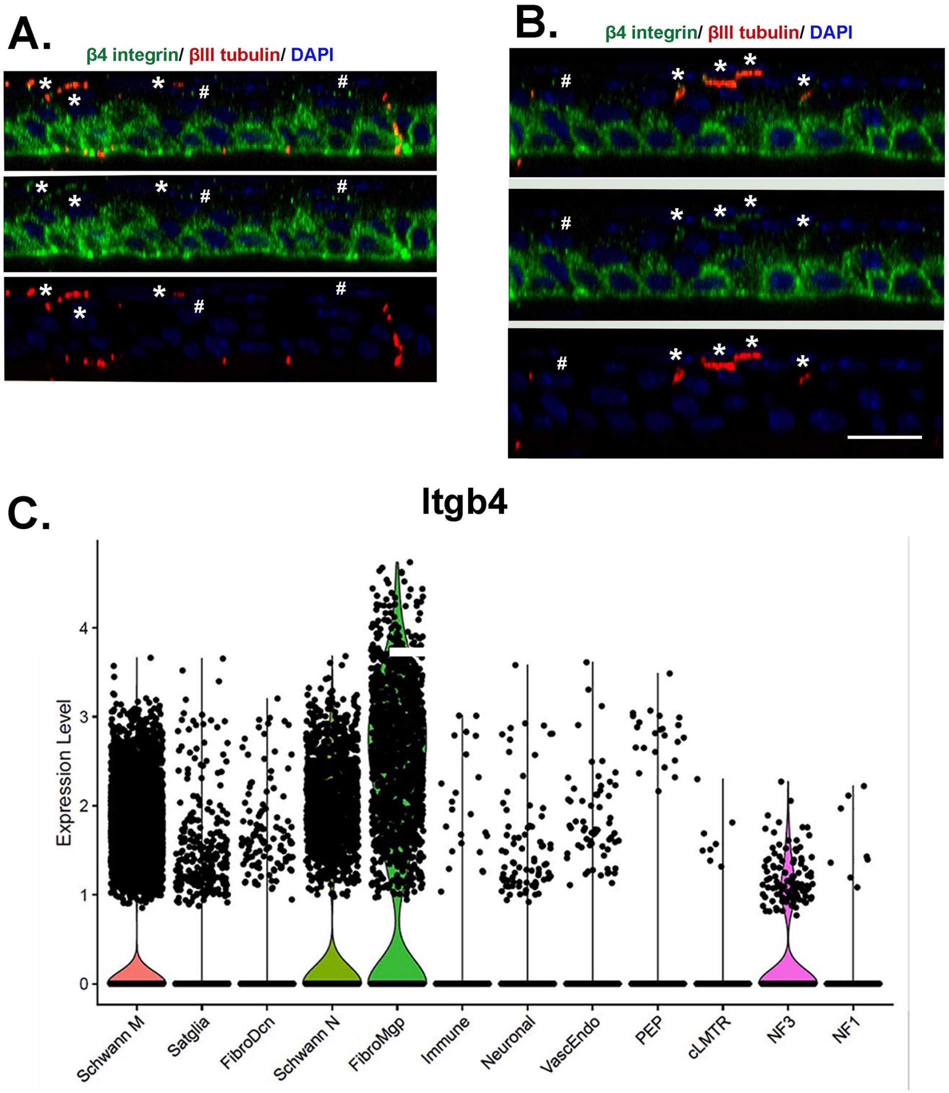

Supplemental Figure 3: β4 integrin is expressed by trigeminal sensory axons.

A and **B** show high resolution confocal images taken from two different corneas 2 weeks after debridement injury stained to highlight the expression of β4 integrin (green) in βIII tubulin+ nerve terminals (red) indicated with white asterisks. The white number symbols show sites where β4 integrin is detected in suprabasal locations without βIII tubulin. **C.** Single nuclei (sn) RNAseq was performed on mouse trigeminal ganglia and data mined to reveal whether mRNA for β4 integrin (Itgb4) was expressed in trigeminal neurons. Data indicate that several neuronal cell types express Itgb4 mRNA. These data can be accessed via the following link: https://singlecell.broadinstitute.org/single_cell/study/SCP2610. Bar is 15 μm.
